# Normative Data for Penile Length in Turkish Newborns

**DOI:** 10.4274/jcrpe.v2i3.107

**Published:** 2010-08-03

**Authors:** Alev Oğuz Kutlu

**Affiliations:** 1 Zübeyde Hanim Maternal Hospital, Pediatric Endocrinology, Ankara, Turkey; +90 532 396 47 78+90 312 437 93 06alevkutlu64@yahoo.com.trNenehatun Caddesi No: 61/3 Gaziosmanpasa, 06430, Ankara, Turkey

**Keywords:** Stretched penile length, newborn, Turkish boys

## Abstract

**Background**: Local normative data for penile size will aid physicians in clinical practice. There are no studies on stretched penile length (SPL) in newborn Turkish boys.

**Objective**: To establish normative data for SPL of newborn Turkish boys and compare these with data from different countries. Methods: 514 newborn Turkish boys, who were not small for gestational age (SGA) or premature, were included in this cross−sectional study. SPLs of the newborns were measured by the same investigator. The correlations between SPL and gestational age, weight, length, and head circumference were evaluated.

**Results**: The 3^rd^ percentile value for SPL was found as 3.00 cm in these Turkish newborns. Positive correlations were shown between SPL and height (r=0.240, p<0.001), weight (r=0.251, p<0.001), and head circumference (r=0.235, p<0.001). Multiple linear regression analysis showed that SPL positively correlated with height and head circumference (p=0.021 and p= 0.042, respectively).

**Conclusions**: This is the largest study on SPL in newborns from our country. This normative data can be used in clinical practice for defining micropenis.

**Conflict of interest:**None declared.

## INTRODUCTION

Size abnormalities of the external genitalia in newborn males and females may be the first and only sign of underlying severe endocrine or genetic disorders (1). Micropenis is the best−known sign of congenital hypopituitarism. It is also seen in Noonan syndrome and Robinow syndrome, as well as in chromosomal abnormalities such as Klinefelter syndrome and Prader−Willi syndrome. Micropenis may be accompanied by undescended testes or hypospadias indicating underlying testicular defects or hormone deficiencies. Micropenis may rarely be seen as a variant in the community due to androgen deficiency developing temporarily in the late stages of pregnancy (2).

Since penis size is reported to vary between different races (1, 2, 3, 4), reference values based on local normative data for penile length are important to avoid overestimation or underestimation. To our knowledge, there are no previous studies from our country on the stretched penile length (SPL) of newborn males.

In this study, we measured the SPL of newborn males born at Zubeyde Hanim Maternal Hospital and evaluated the correlation of SPL with anthropometric parameters at birth such as head circumference, length, and weight. We then compared the reported SPL values from various ethnic groups and races with our own results.

## METHODS

**Study group**

Penile measurements were performed within the first 24 hours of life in 514 newborn males born by spontaneous vaginal delivery. Written informed consent was obtained from the parents. The study was approved by the hospital ethics committee.

All measurements were performed by the author (A.O.K.) in an examination room, where the temperature was held constant at 23−24°C. The newborns were examined in supine position and a nurse held the newborn’s legs in a flexed position. A spatula was placed at the base, along the dorsal aspect of the penis, and maximal pressure was applied to the fatty tissue on the pubic ramus. Traction was applied to the penis to the point of increased resistance, and the projection of the tip of the glans was marked on the spatula. The distance between the base of the penis and the tip of the glans was measured using a digital caliper. Two consecutive penile measurements were made, and the mean value was calculated.

Gestational age was calculated from the first day of the mother’s last menstrual period, if the mother had regular menstrual cycles and was certain of her menstrual history. Newborns whose mothers were not able to give an exact date for their last menstrual period or did not have regular menstrual cycles were excluded.

Length, weight, and head circumference of the patients were also measured. Length was measured in the supine position, and weight was measured with a scale sensitive to 10 g.

Only infants who were not small for gestational age (SGA) or premature and who had a birth weight of 2500 g or more were included in the study. Patients with an external genital anomaly, such as undescended testes or hypospadias, or those with webbed penis or a hidden structural penile abnormality were excluded. All children were uncircumcised.All measurements were performed by a single examiner.The study was approved by the hospital ethics committee. All parents provided written informed consent prior to the study.

**Statistical Analysis**

Data analysis was performed using the software package SPSS 13.0 for Windows. Continuous variables were expressed as mean±standard deviation (SD). Categorical variables were expressed as percentages. The Pearson’s correlation coefficient was used to determine the presence of a linear relationship between SPL measurements and gestational age, length, weight, and head circumference. The multiple linear regression analysis was used to explore the factors that have an influence on SPL measurements. The 3^rd^, 5^th^, 10^th^, 25^th^, 50^th^, 75^th^, 90^th^, 95^th^ and 97^th^ percentile values were calculated for SPL measurements. The ±2.5 SD limits were determined for SPL measurements according to gestational age. The least mean squares (LMS) method was applied with penalized likelihood to develop reference percentile curves. The LMS method summarizes the changing distribution with three curves representing the mean, variability factor and distortion. These three curves are intersected in a non−cubic way using the maximum penalized likelihood method. The necessary consistency is realized using smoothing parameters or the equivalent degree of freedom (5).

The coefficient of variation (CV) and the interclass correlation coefficient (ICC) were measured for the 1^st^ and 2^nd^ SPL levels of 22 randomly selected cases. A p−value less than 0.05 was accepted to be statistically significant.

## RESULTS

The mean pregnancy duration for the study subjects in our study was 277.63±7.00 days. The mean values in newborn subjects for length was 49.95±1.90 cm, for weight 3.31±0.40 kg, for head circumference 34.88±1.12 cm, and for SPL 3.77±0.35 cm.

We found no correlation between SPL and gestational age (r=0.051, p=0.248).

The results of the univariate correlation test revealed a positive correlation between SPL and body length (r=0.240, p<0.001), weight (r=0.251, p<0.001), and head circumference (r=0.235, p<0.01). Multiple linear regression analysis, performed to determine the relationship between these variables and SPL, showed a continuning effect of length and head circumference on SPL (p=0.021 and p= 0.042, respectively), but no effect of weight.

The following equation was formulated to determine the estimated (corrected) penile length of the cases according to head circumference and length:

SPL=1.128 + 0.023 x(length)+0.035x(head circumference)

The 95% confidence intervals for regression coefficients related to length and head circumference were 0.003−0.043 and 0.001−0.068, respectively. Figure 1 and 2 show SPL percentiles by length and head circumference of the newborns.

The coefficient of variation was 0.3%, and the intra observer correlation level (95% confidence interval) was 99.2% (98.1%−99.7%) for penile measurements.

**Figure 1:**
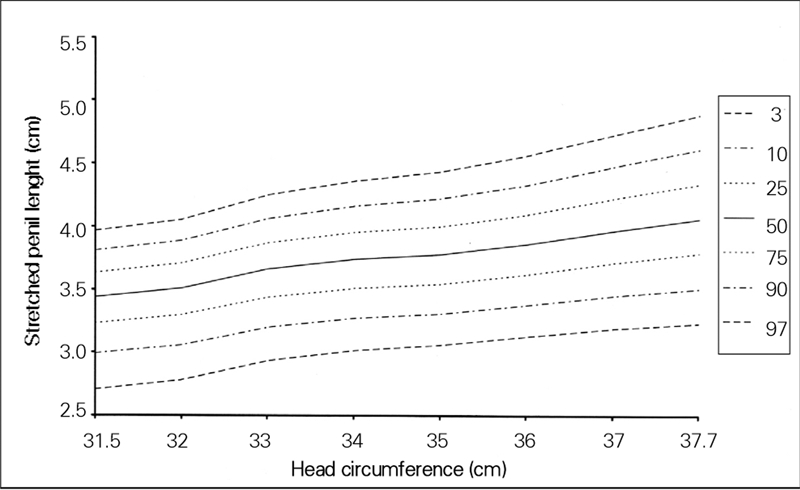
Percentile curves for stretched penile length by length for newborn Turkish boys

**2:**
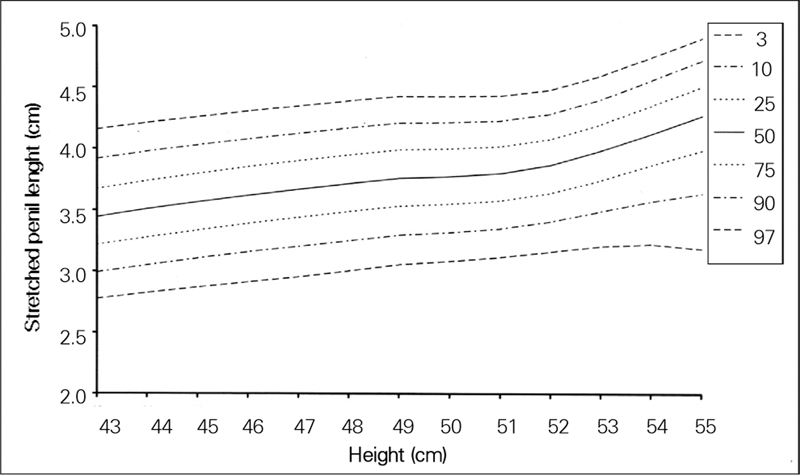
Percentile curves for stretched penile length by head circumference for newborn Turkish boys

## DISCUSSION

Both male and female external genital organs develop from the genital tubercle in the intrauterine period (1). The human chorionic gonadotrophin−testis axis promotes penis development from the genital tubercle at 8^th^ to 14^th^ weeks of pregnancy. Penile growth is fastest (4 cm/year) between the 20^th^ week of gestation and birth due to the rapid development of the hypothalamic−pituitary−testicular axis (6). There is also a rapid increase in penile length in parallel to the testosterone surge in mini−puberty in the first 3 months after birth (7). Subsequently, there is an increase of 1−2 cm in total until the ages of 6−7 years (8).

SPL has been considered to be a gold standard measurement since 1942 after an article by Schonfeld (9). However, study results can be influenced by factors like erect penis or cold environment (7). Differences in measurements as great as 0.5 cm have been reported among different investigators (7). Two studies on South Indian populations showed great differences in penile length at birth ranging from 2.31 to 3.57 cm (10, 11). In our study, all measurements were repeated twice by the same investigator at constant room temperature, and the mean of the two measurements was calculated. Infants with structural penile abnormalities, such as webbed or hidden penises, were excluded. The intra−observer reliability coefficient was 99.2% (98.1%− 99.7%) indicating that the measurements were consistent and that the study has high reliability.

In this study, which did not include SGA or preterm babies, we found no correlation between SPL and gestational age. However, Fok et al (12), who also measured SPL only in term babies, reported increased SPL with gestational age. We believe that the unequal distribution of our patients by gestational week may have affected our statistical results. We only included appropriate for gestational age (AGA) infants into the study and found no correlation between SPL and birth weight. Similarly, Lian et al (2) found no influence of birth weight on SPL and therefore, postulated that references used for AGA babies could also be applied to SGA babies. In contrast to the literature, we found a significant correlation only between SPL and head circumference and length, and developed percentile curves for these (Figures 1 and 2). We assume that there are some common growth factors influencing the development of the penis, length and head circumference in the intrauterine period. Although we did not come across any studies on this relationship, the fact that Laron syndrome with insulin−like growth factor−1 (IGF−1) deficiency presents with short stature in addition to decreased head circumference and decreased penile length indicates that IGFs or other growth factors are possibly responsible for all three (13, 14).

Various studies have reported an interracial difference for SPL (1, 12). For example, SPL is shorter in Chinese children than in Caucasians or children of other Asian countries. Chinese children can be diagnosed to have micropenis if they are not evaluated by their own references (12). Studies on SPL published prior to year 2000 mostly concerned non−Asian babies. The studies by Feldman (3) and Lian (2) included Asian babies and reported similar results (3.1±1.1 cm and 3.6±0.4 cm, respectively). A study from Israel found an SPL value of 3.5 cm±0.4 cm in 100 term newborns (4). Our mean SPL value was 3.77±0.35 cm and was similar to the Indian babies in Lian's study. These studies make it clear that there is an interracial difference for newborn SPL values and that each race should therefore have its own normal values.

Micropenis is traditionally defined as an SPL value below the 10^th^ percentile (12). This definition is not specific, as 10% of all newborn males receive a diagnosis of micropenis with this definition and have to undergo unnecessary advanced tests. Some authors suggest that micropenis be defined as an SPL value less than 2.5 SD of the mean (15). The 2.5 SD was 2.9 cm and the 10^th^ percentile 3.3 cm in this study, thus, there was a difference of 0.5 cm between these two definitions. We believe that micropenis should be defined as an SPL value below the 3rd percentile or −2 SD (3 cm and 3.02 cm, respectively in our study), similar to the definition of short stature.

In conclusion, this is the largest study on SPL in newborns from our country. Although the study reliability was increased by having the same investigator conduct the study, larger study groups representing the Turkish boys are necessary. A consensus obviously needs to be developed for the definition of micropenis.

**Figures 1:**
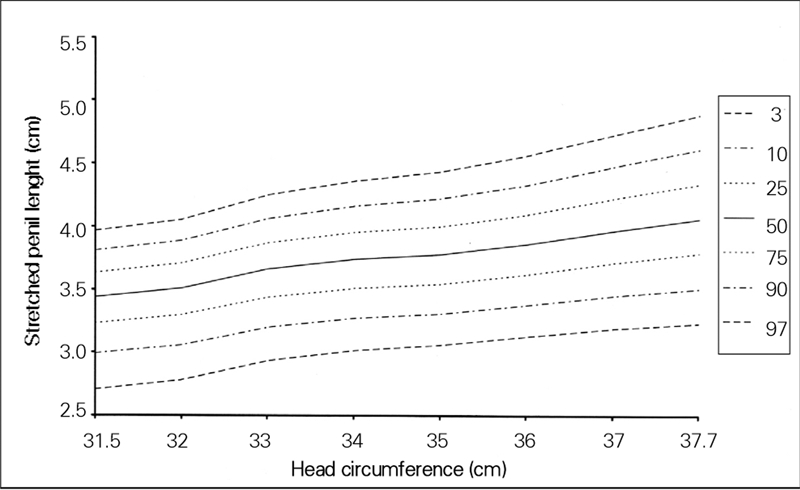
Percentile curves for stretched penile length by length for newborn Turkish boys

**2:**
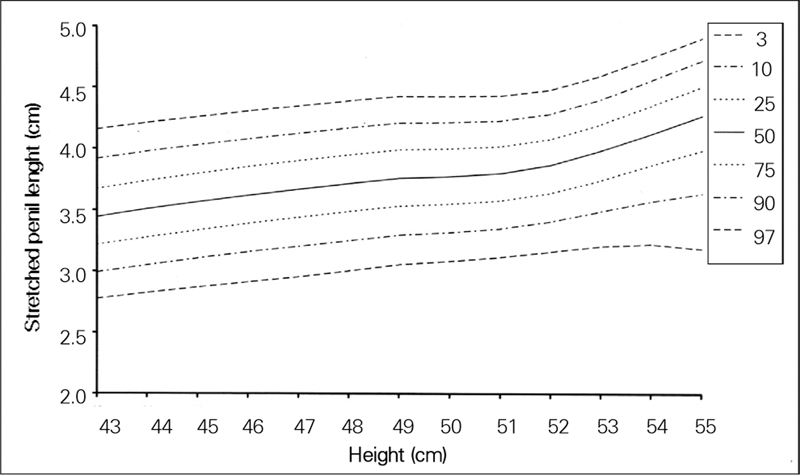
Percentile curves for stretched penile length by head circumference for newborn Turkish boys
